# Travel Medicine, 2nd Edition

**DOI:** 10.3201/eid1503.081591

**Published:** 2009-03

**Authors:** Lin H. Chen

**Affiliations:** Mount Auburn Hospital, Cambridge, Massachusetts, USA; Harvard Medical School, Boston, Massachusetts, USA

**Keywords:** Travel medicine, epidemiology, disease prevention, book review

The second edition of this valuable textbook highlights a dynamic specialty that integrates many disciplines. Edited by 5 internationally renowned leaders in travel medicine, the book assembles a fine group of authors and chapters. The print version is hard cover and comes with online access that is easy to use and fully searchable. Both versions include useful maps, tables, and figures, which can be exported from the online version to PowerPoint presentations to assist in teaching. The online version also links to abstracts of references, allowing readers easy access to the abstracts simply by clicking on linked references.

The book consists of 57 well-organized chapters that begin with fundamental topics such as epidemiology, travel clinic management and resources, and basic disease prevention. The chapters then progress to more specialized topics such as special hosts (e.g., immunocompromised travelers), special itineraries (e.g., expatriates and persons on expeditions), health problems while traveling, and posttravel care. This edition also features a new chapter on cruise ship travel. The chapters vary greatly in length and content: chapters on jet lag and sun-associated problems are brief; chapters on topics such as immunization of healthy adults are detailed and lengthy (i.e., 36 pages). The immunization chapter covers a large amount of material that is challenging because it addresses international variations in vaccine licensure and guidelines. For instance, tick-borne encephalitis vaccine and cholera vaccine, which are described in this chapter, are available in Europe but not in the United States. However, the global nature of travel medicine necessitates practitioners’ knowledge of these vaccines.

As the field of travel medicine matures and its evidence base increases, topics such as environmental aspects of travel, psychological aspects of travel, and travel injuries are gaining greater recognition. If a new edition is considered, additional topics that would be helpful to clinicians include travelers’ precautions for eye care (especially regarding certain environmental exposures), breastfeeding among travelers (currently an abbreviated section in a chapter that discusses pregnant travelers), and collaboration among practitioners of travel medicine and public health professionals. Specific policies and procedures for travel clinic management could also address the triage of posttravel illnesses.

This book is a comprehensive reference on travel medicine. It is rich in information, pleasant to read, and practical. It has been updated to recommend current best practices in travel medicine. The editors have assembled an excellent textbook, and I recommend it enthusiastically to health professionals interested in this growing specialty.

